# Impacts of climate change on the global spread and habitat suitability of *Coxiella burnetii*: Future projections and public health implications

**DOI:** 10.1016/j.joclim.2025.100442

**Published:** 2025-04-15

**Authors:** Abdallah Falah Mohammad Aldwekat, Niloufar Lorestani, Farzin Shabani

**Affiliations:** College of Arts and Sciences, Qatar University, P.O. Box 2713, Doha, Qatar

**Keywords:** Climate change, *Coxiella burnetii*, Species distribution models (SDMs), Future climate projections, Global circulation models (GCMs)

## Abstract

**Introduction:**

*Coxiella burnetii*, an intracellular zoonotic bacterium, affectsing various livestock and wildlife species and poses significant risks to human health. This study aims to assess how climate change could impact the global distribution and habitat suitability of *Coxiella burnetii*, the pathogen responsible for Q fever.

**Materials and methods:**

An ensemble species distribution modelling approach, integrating regression-based and machine-learning algorithms (GLM, GBM, RF, MaxEnt), was used to project habitat suitability (Current time and by 2050, 2070, and 2090). Climate variables were obtained from five global circulation models (GCMs) under two climate change scenarios (SSP2-4.5 and SSP5-8.5). The study evaluated the models’ performance using the area under the curve (AUC) and true skill statistics (TSS).

**Results:**

Results show that under current climate conditions, *C. burnetii* is widespread across regions like North and South America, Europe, and parts of Africa, Asia, and Australia. Future projections indicate a northward shift in habitat suitability, especially under the severe SSP5-8.5 scenario, with significant expansions into Russia, northern Europe, and Canada. Conversely, regions in South America, Africa, and Australia may see declines in suitable habitats. By 2090, a 44.56 % (range: 33–57.9 %) across the models, increase in suitable habitat is predicted, accompanied by a 27.66 % (range: 22.4–31.7 %) loss of current habitats.

**Discussion:**

Findings indicate that temperature seasonality and precipitation of the driest month are the most influential climatic variables shaping the distribution of *C. burnetii*. These results underscore the importance of climate variability in influencing the pathogen's global distribution and highlight the critical role of environmental factors in predicting future habitat shifts.

**Conclusion:**

The study highlights the profound impact climate change could have on the global distribution of *C. burnetii*. It underscores the need for proactive public health strategies in emerging high-risk areas and emphasizes the importance of mitigating risks in regions experiencing habitat declines. These findings offer valuable insights for public health planning and livestock management under future climate scenarios. In interpreting these results, it is important to consider modelling uncertainties, including assumptions and data limitations.

## 1 Introduction

*Coxiella burnetii* (*C. burnetti*) is a zoonotic pathogen responsible for Q fever, a disease that affects animals and poses a significant risk to public health [1]. It is particularly notable for its resilience in various environmental conditions and its capacity to spread rapidly across regions. Its epidemiological pattern consists of sporadic occurrences, endemic circumstances, and unexpectedly large outbreaks worldwide [[2], [3]–4]. *C. burnetii C. burnetii* is an intracellular Gram-negative bacterium belonging to the phylum Proteobacteria, family Coxiellaceae, class Gammaproteobacteria [5]. Although *C. burnetii* was previously considered a member of the genus *Rickettsia*, a pleomorphic bacterium, it is now included in the order *Legionellales* [6,7].

The bacteria are present across various animal species [2] including goats, sheep, cattle, horses, swine, camels, water buffalo [8,9], birds [10], and arthropods, mainly ticks [11]. *C. burnetii* can be found in feces, milk [12], urine, and birth products of mammals [7,13,14]. While the infection is often asymptomatic in livestock, it can cause miscarriages, stillbirths, early deliveries, the birth of weak offspring, decreased fertility, endometritis, and infertility [15,16].

Although living close to livestock is a common risk factor for infection, more than two-thirds of Q fever outbreaks globally have occurred in communities outside typical high-risk areas, where many people had no direct interaction with livestock [17,18]. Approximately 60 % of Q fever cases in humans are asymptomatic [19]. However, in clinically evident cases, the disease can result in stillbirths or spontaneous miscarriages in pregnant women, flu-like symptoms in the lower respiratory tract and chronic infections, which account for around 2–5 % of acute cases. As *C. burnetii* can persist within the host for extended periods, its progression may cause chronic conditions such as osteomyelitis, hepatitis, interstitial lung disease, endocarditis, vascular disorders, and chronic fatigue syndrome [20,21]. Therefore, individuals at higher risk of infection include veterinarians, farmers, pregnant women, immunocompromised individuals, and patients with valvular disease [22]. The global mortality rate of Q fever varies, with lethality reported at <3 % in Spain [18] and the USA [23] and as high as 10 % in California [24]. Q fever has been reported worldwide, with the highest numbers documented in Australia, the UK, France, and the USA [17]. The most significant outbreak happened in The Netherlands between 2007 and 2010, which affected over 3500 humans, which about 20 % of reported cases required hospitalization for acute illness [25]. Q fever outbreaks do not exhibit a consistent pattern; most outbreaks are sporadic and temporary. Some outbreaks have been explosive, affecting hundreds of people briefly, while others involved only a few cases over an extended timeframe [17].

Q fever spreads through direct and indirect transmission mechanisms [26]. In humans, indirect transmission occurs primarily via aerogenic routes, where inhalation of contaminated aerosols from infected animal products such as milk, feces, birth materials, and urine can lead to infection. As few as 10 bacteria in these aerosols can cause illness [5,27]. Direct transmission occurs through the consumption of contaminated raw milk and dairy products [28] or or through contact with infected animals during abortion and parturition, **as** well as through exposure to contaminated materials such as milk, feces, semen, and urine [29]. In animals, the main route of *C. burnetii* infection is the inhalation of infectious aerosols or dust [30]. Ticks are also one of the major factors in the persistence of the pathogen in animal populations [31,32].

Beyond its biological characteristics and epidemiological patterns, the survival and spread of *C. burnetii* is significantly influenced by environmental factors. Environmental factors such as wind and landscape can further enhance the spread of aerosols, contributing to outbreaks [6,28]. Dry conditions with low precipitation often facilitate the persistence of spores and wind-borne transmission. Additionally, temperature fluctuations can affect the pathogen's survival, with extreme heat or cold potentially reducing its viability over time. *C. burnetii* and its spore-like forms exhibit remarkable resilience. Under favorable conditions, they can survive for weeks to months outdoors [21,33]. As climate change continues to alter environmental conditions globally, the distribution and habitat suitability of zoonotic pathogens like *C. burnetii* are expected to shift. The increasing frequency of extreme weather events, changes in temperature, and altered precipitation patterns provide favorable conditions for the pathogen's spread. Given the critical role of environmental factors in *C. burnetii*'s survival and spread, understanding these drivers in diverse geographic and environmental contexts is crucial.

Habitat suitability modelling offers a robust framework for analyzing how environmental factors influence the distribution of *C. burnetii*. This approach is widely used to predict the species' potential distribution under current and future environmental conditions. This method identifies areas where environmental factors, such as temperature, precipitation, and other variables, align with the ecological requirements of the species or pathogen being studied. Habitat suitability modelling has become an essential tool in ecology, biogeography, and conservation [[34], [35], [36]–37]. Species Distribution Models (SDMs) predict where species are likely to occur based on their environmental preferences, providing valuable insights into global distribution patterns and the potential impacts of environmental changes on species distributions [[38], [39], [40]–41]. SDMs integrate species occurrence records with environmental covariates derived from the locations where the species have been observed [42]. Previous studies on *C. burnetii* distribution have predominantly focused on specific countries and often relied on a single modelling approach such as Maxent [43,44]. However, this study utilizes a comprehensive, ensemble-based approach. By integrating multiple models and future climate projections, our study provides a more robust prediction of the global spread of *C. burnetii* under different climate scenarios. Furthermore, we utilized five different global circulation models (GCMs) and two shared socio-economic pathways (SSP2-4.5 and SSP5-8.5) to address uncertainties in future climate projections. This paper aims to *i*) identify potential high-risk areas for *C. burnetii* infection worldwide and *ii*) evaluate how climate change may influence the pathogen's global distribution in the future. Our findings offer critical insights for public health planning, livestock management, and strategic resource allocation in response to evolving pathogen dynamics under climatic change conditions.

## 2 Materials and methods

### 2.1 Species geographical data

**Fig. 1:**
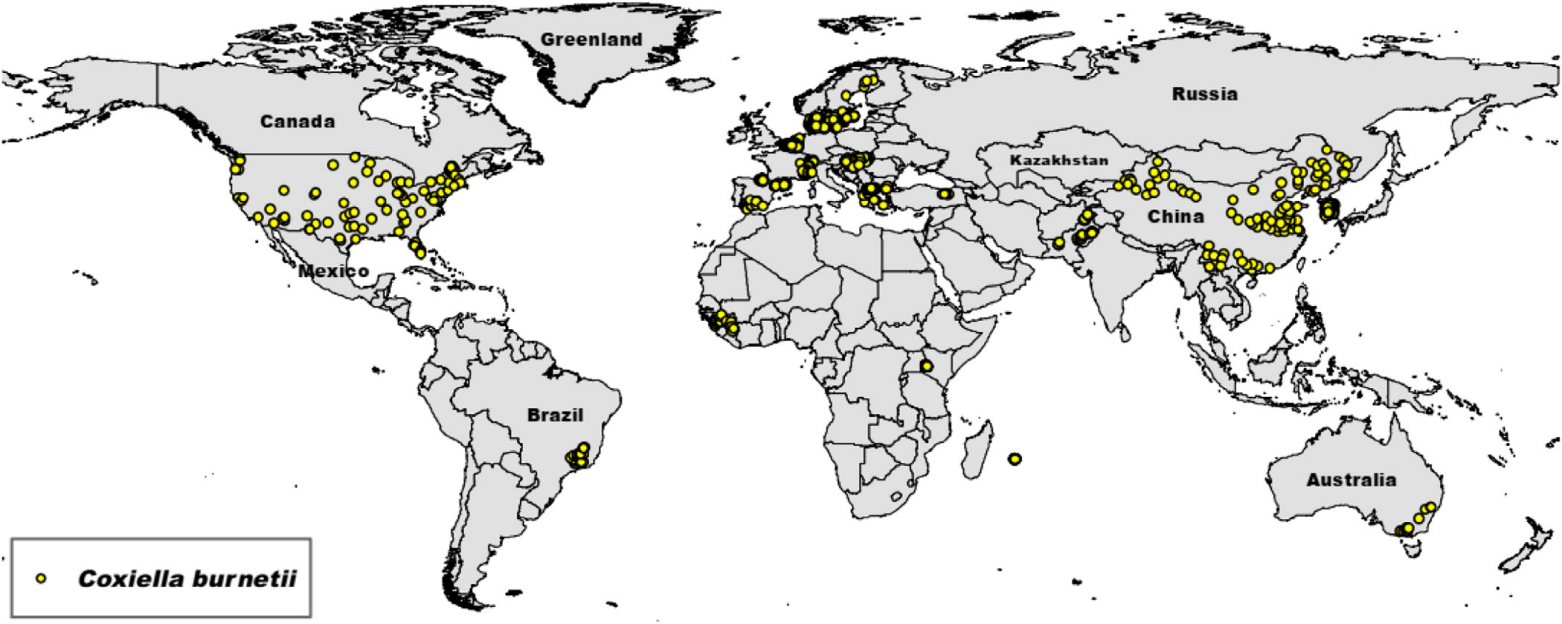
The global geographic distribution of *C. burnetii* occurrence points (yellow dots) used in species distribution modelling. These points reflect data collected from the Global Biodiversity Information Facility (GBIF) and literature sources.

To collect a comprehensive dataset on the global distribution of *C. burnetii* species, presence points were gathered from diverse sources, including the Global Biodiversity Information Facility (GBIF) [45] and scientific literature review (details of literature-derived occurrence points are provided in the Table S1). To ensure data accuracy from the GBIF dataset, we used the package “Coordinate Cleaner” [46] in the R environment. Additionally, to reduce potential spatial autocorrelation [47], duplicate points within a 5-km radius were removed. Accordingly, 1001 presence points remained for the SDM analysis (Fig. 1). It should be noted that while the above-mentioned steps reduce bias, residual issues related to data availability and geographic sampling gaps remain a limitation.

### 2.2 Climatic variables

In this study, climatic variables were obtained from the WorldClim database at 2.5 arc-minutes (∼4.5 km) resolution, representing long-term monthly averages over the baseline period (1970–2000). We also included projections for three future timeframes: 2050 (2040–2060), 2070 (2060–2080), and 2090 (2080–2100) [48]. To project future changes up to 2100, we employed climate models from the Coupled Model Intercomparison Project Phase 6 (CMIP6), (see O'Neill, Tebaldi [49] for details). These models simulate growth trends based on future Shared Socio-economic Pathways (SSPs). Two (SSP) scenarios were selected for this study. SSP2-4.5 represents intermediate carbon emissions, moderate development and uneven global progress, with a projected temperature increase of approximately +2.7 °C by 2100. In contrast, SSP5-8.5 reflects the highest emission levels, driven by rapid global economic growth and heavy reliance on fossil fuels, leading to a projected temperature increase exceeding +4.4 °C by the end of the century [49,50]. To simulate future climatic conditions, five global circulation models (GCMs) were employed: ACCESS-CM2, IPSL-CM6A-LR, MIROC6, MPI-ESM1–2-LR, and UKESM1–0-LL. This multi-model approach helps mitigate the inherent uncertainties in climate predictions. By incorporating diverse climate scenarios, we gained a more robust and comprehensive understanding of potential future climates, which is crucial for assessing the species' future suitability [51].

To address potential multicollinearity among variables, we calculated the Variance Inflation Factor (VIF) using the "usdm" package [52] in the R environment. Variables with VIF values greater than 6 were excluded from the analysis. After filtering, we selected 8 bioclimatic variables for the species distribution modelling (SDM) analysis. Details of the selected variables are provided in Table 1

**Table 1:** Definitions and details of the selected bioclimatic variables used in the study (Source: WorldClim).

Variable	Code	Definition	Unit
Mean diurnal range	bio2	The mean of the monthly temperature ranges (monthly maximum minus monthly minimum)	°C
Temperature seasonality	bio4	Standard deviation of monthly mean temperatures multiplied by 100.	°C
Mean temperature of the wettest quarter	bio8	Average temperature during the wettest three-month period (quarter).	°C
Precipitation of the wettest month	bio13	Total precipitation during the single wettest month.	mm
Precipitation of the driest month	bio14	Total precipitation during the single driest month.	mm
Precipitation seasonality	bio15	Coefficient of variation of monthly precipitation (standard deviation divided by the mean).	%
Precipitation of the warmest quarter	bio18	Total precipitation during the warmest three-month period (quarter).	mm
Precipitation of the coldest quarter	bio19	Total precipitation during the coldest three-month period (quarter).	mm

### 2.3 SDM analysis

We evaluated the habitat suitability of *C. burnetii* using one regression-based method: generalized linear models (GLM), and three machine-learning algorithms: generalized boosting model (GBM), random forest (RF), and maximum entropy (MaxEnt), with the package “biomod2” [53] in R v 4.3.2.

The ensemble model was constructed using weighted averaging based on the predictive performance (AUC and TSS) of each algorithm. Utilizing multiple models and integrating them into an ensemble model could enhance the accuracy of model predictions and reduce the uncertainty associated with relying on a single model [[54], [55], [56]–57]. As a requirement for the biomod modelling framework, we generated a random sample of 10,000 background points, ensuring they did not overlap with presence points, and employed a 5 fold cross-validation to create training and test datasets. In this approach, the presence and background records were randomly divided into 5 folds, and each time one-fold was left out for testing the sample, and other folds were used for training the model.

The models' performance was evaluated using the area under the receiver operating characteristic curve (AUC) [58], which ranges from 0.5 to 1 (values close to 1 indicating perfect discrimination), and the true skill statistic (TSS) metrics, ranging from −1 to +1 (where 1 indicates perfect classification accuracy) [59]. To understand the relative contribution of each climatic variable to the distribution of *C. burnetii*, the associated scores were averaged across all models. This process allowed us to identify the importance of each variable.

Following the completion of the current ensemble model, we projected the predicted potential distribution model into the future climatic scenarios, all implemented in R environment v 4.3.2. We employed ensembles to generate combination maps for future distribution [55]. Ultimately, a series of projection maps for the years 2050, 2070, and 2090, based on each SSP were produced.

We compared the current and future suitability to investigate changes in *C. burnetii'*s distribution. To achieve this, we converted the continuous habitat suitability maps generated by species distribution models (SDMs) into binary maps (suitable/unsuitable) using a threshold that maximizes the sum of sensitivity and specificity [[60], [61]–62]. Based on the binary maps, we calculated three indices to reflect the impact of climate change on the distribution of *C. burnetii:*

• **Stable:** The count of pixels that remain suitable in both current and future projections.
• **Loss:** The count of pixels that are currently considered suitable but are projected to become unsuitable in the future.
• **Gain:** The count of pixels that are currently considered unsuitable but are projected to become suitable in the future.

These indices allowed us to quantify the extent of habitat shifts, losses, and gains under different scenarios and timeframes. To account for variability in climate projections, we calculated the mean percentage gain or loss across five global circulation models (GCMs).

## 3 Results

### 3.1 Global distribuion of *C. burnetii*

The occurrence points of *C. burnetii* indicate a high occurrence in the USA, particularly in the eastern regions. Significant occurrence records were also identified in Europe, China, Guinea, Brazil, and Pakistan. Additionally, our literature review provided presence points in Kenya, Mauritius, Turkey, and southwestern Australia. However, based on the collected occurrence data, no documented records of *C. burnetii* are available for specific locations in Canada, most of the Middle East, the Gulf region, large parts of Africa, Russia, or India (Fig. 1). It is important to note that these patterns reflect the available data used in this study and do not necessarily indicate the species' true distribution.

### 3.2 Model performance

**Fig. 2:**
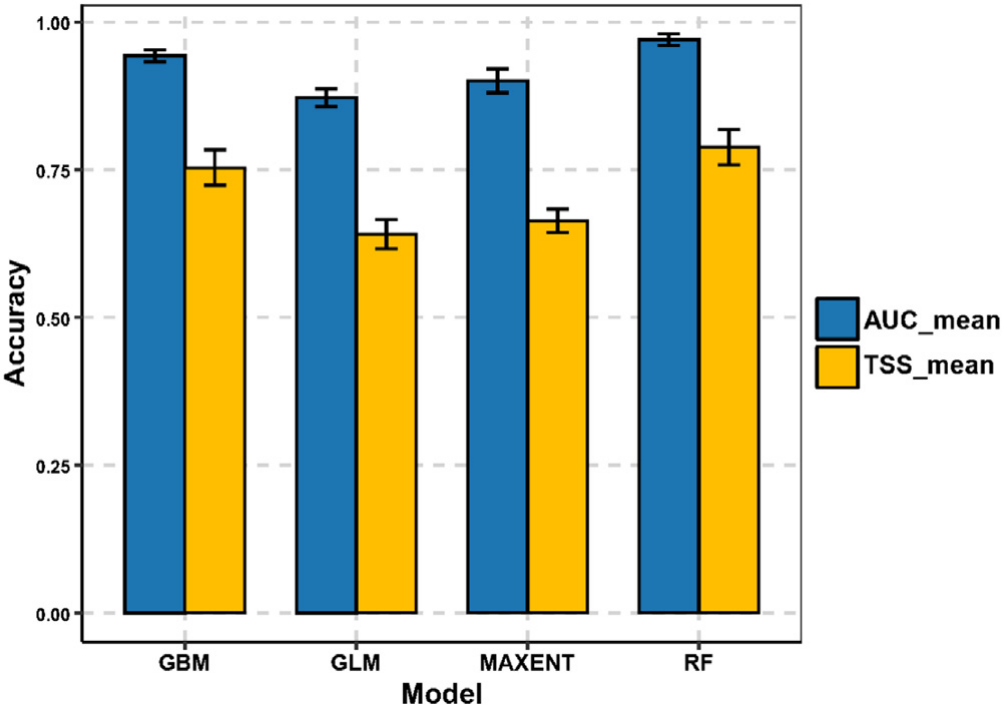
Mean AUC and TSS values for the GBM, GLM, MAXENT, and RF models used in species distribution modelling of *C. burnetii*. The blue bars represent the Area Under the Receiver Operating Characteristic Curve (AUC), while the yellow bars indicate the True Skill Statistic (TSS).

The results of SDMs for *C. burnetii* showed excellent predictive performance. RF achieved the highest predictive performance (AUC = 0.97 and TSS = 0.789), followed by GBM (AUC = 0.943 and TSS = 0.754) and MaxEnt (AUC = 0.901 and TSS = 0.663). Lastly, GLM yielded the lowest predictive performance (AUC = 0.872 and TSS = 0.641) (Fig. 2). It should be noted that while AUC and TSS are widely used and provide robust measures of model performance, they have limitations. AUC does not account for the prevalence of species, and TSS may overestimate accuracy in cases of imbalanced data. These metrics were selected for their simplicity and effectiveness in comparative studies, but future work could integrate additional metrics, such as sensitivity-specificity balance or Kappa statistics, for a more comprehensive evaluation.

### 3.3 The comparative importance of the climatic variables

The importance of each climatic variable was determined by averaging the relative contribution scores across all algorithms used in the SDMs, with higher scores indicating greater predictive importance. Analyses of relative predictive variables revealed that temperature seasonality (bio4) was the most important variables in predicting the distribution of *C. burnetii*, followed by precipitation of the driest month (bio14) and mean temperature of the wettest quarter (bio8) (Table 2).

**Table 2:** Mean and standard deviation (SD) of the relative importance of the climatic variables in predicting the distribution of *C. burnetii*. Higher mean values indicate a greater contribution of the variable to the predictive models.

Predictor	Mean	SD
Temperature seasonality (bio4)	0.37	0.05
Precipitation of the driest month (bio14)	0.2	0.17
Mean temperature of the wettest quarter (bio8)	0.14	0.03
Precipitation of the coldest quarter (bio19)	0.1	0.04
Precipitation of the warmest quarter (bio18)	0.07	0.05
Precipitation seasonality (bio15)	0.05	0.05
Precipitation of the wettest month (bio13)	0.05	0.05
Mean diurnal range (bio2)	0.02	0.02

### 3.4 Current and future habitat suitability of *C. burnetii* under climate change scenarios

**Fig. 3:**
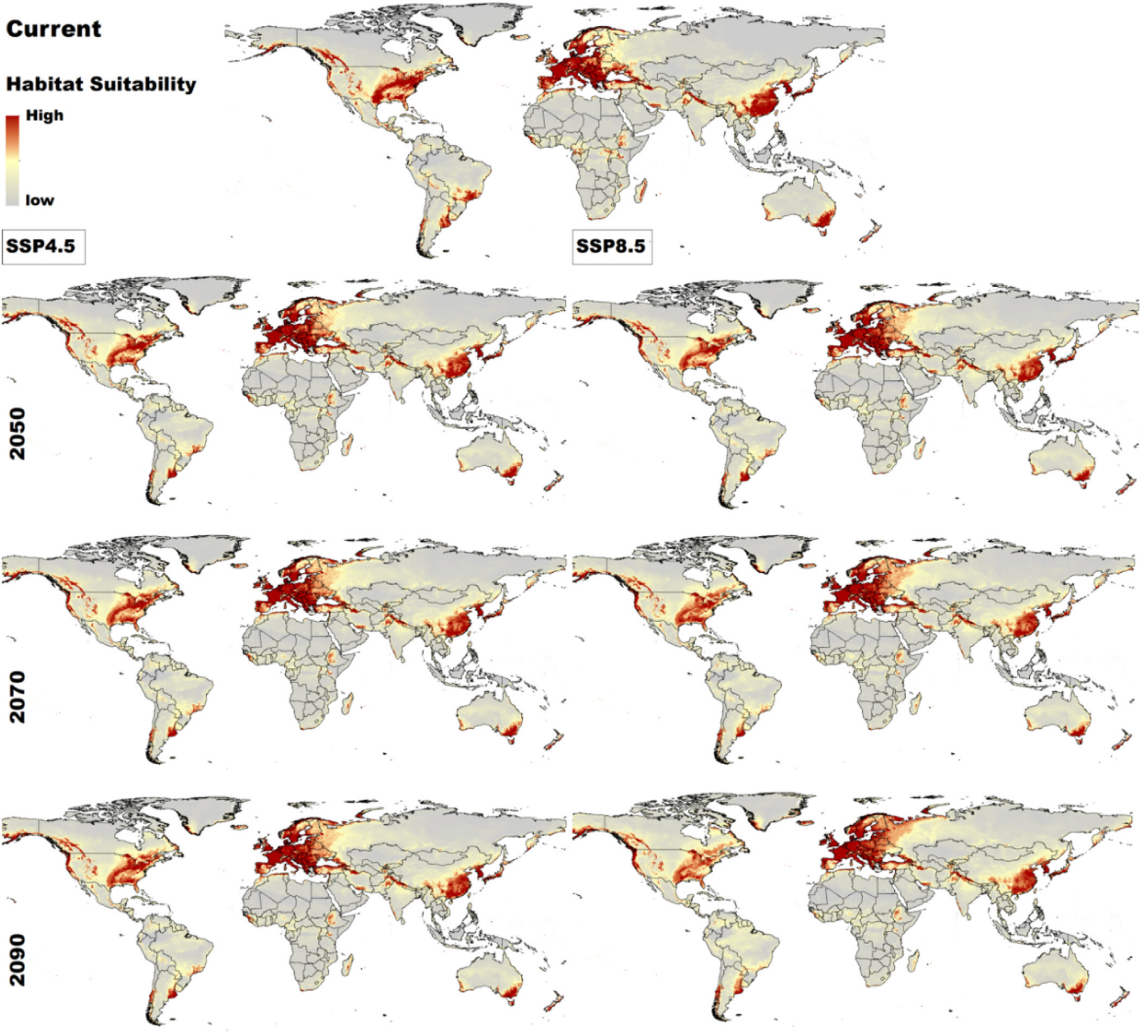
Predicted habitat suitability for *C. burnetii* under current conditions and future climate change scenarios (SSP2-4.5 and SSP5-8.5). Areas of high suitability are represented in dark red. Regions with lower habitat suitability (indicated by lighter shades in Figure 3) include areas across Africa, South Asia, and the Middle East, such as Saudi Arabia, Syria, and Jordan.

Under current climatic conditions, regions with high habitat suitability (represented by dark red areas in Fig. 3) for *C. burnetii* include eastern and central North America, parts of South America (notably Brazil, Argentina, and Chile), central and east Africa (including Kenya, Ethiopia, and Ghana), most of Europe extending into Turkey, regions north of Iran and Pakistan, as well as parts of southern China, Japan, southeastern and southwestern Australia, and New Zealand. Regions with lower habitat suitability (indicated by lighter shades in Fig. 3) include countries such as Saudi Arabia, Syria, and Jordan.

Our projections indicated that by 2050, particularly under the severe SSP5-8.5 scenario, suitable areas in Europe will gradually shift northward, extending into Russia. A similar northward shift is observed in Canada. By 2070 and 2090, this trend becomes more pronounced, with further expansion into northern Europe, Russia, and Canada. These shifts are more significant under SSP5-8.5 compared to SSP2-4.5. Most regions in Europe, North America, and China are projected to remain stable and highly suitable for *C. burnetii* over time, although some fluctuations are expected in these areas. In contrast, certain regions, particularly in South America (notably Brazil), parts of Africa, and Australia are projected to experience a decline in suitable habitats, especially under the SSP5-8.5 scenario.

### 3.5 Shifts in habitat suitability for *C. burnetii*

**Fig. 4:**
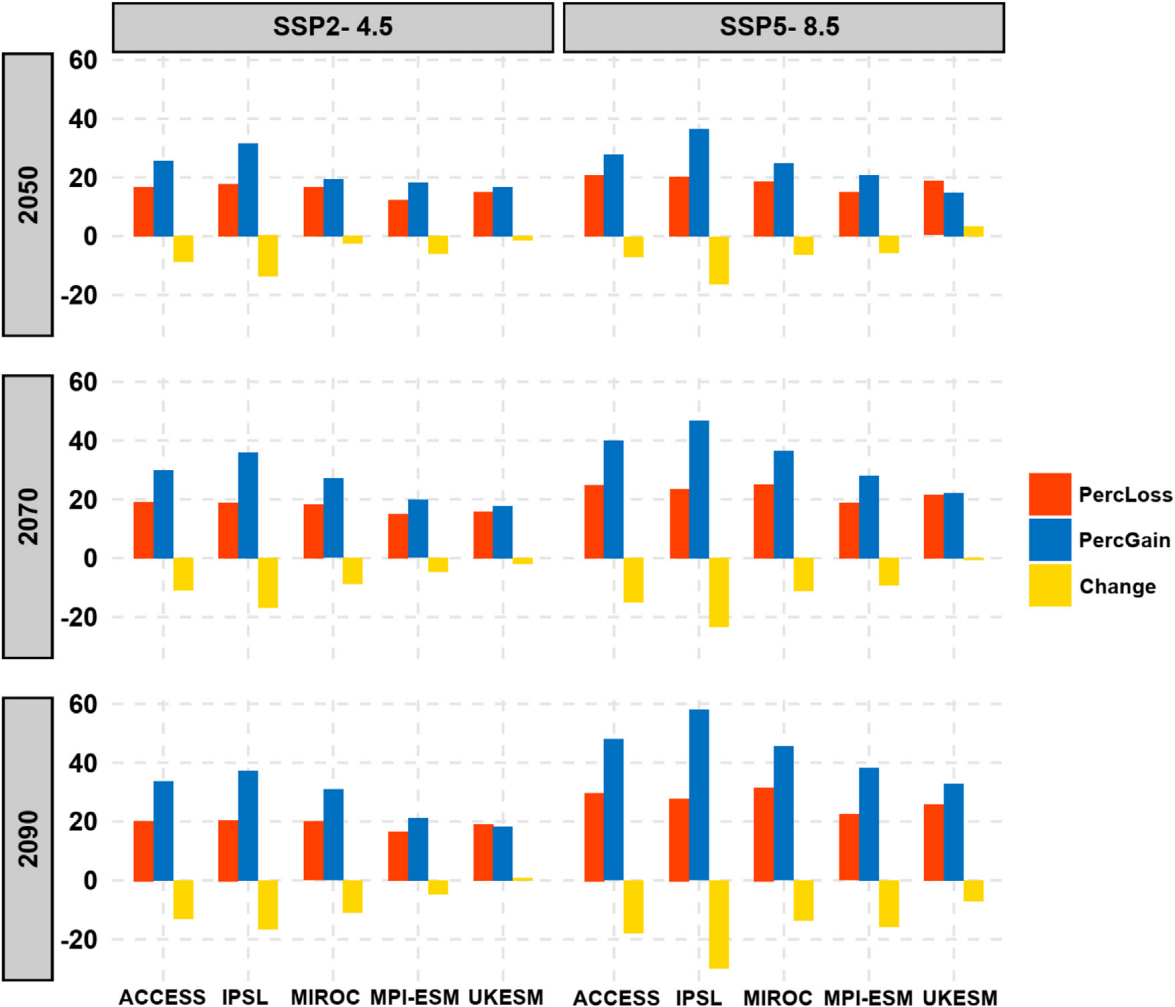
Model-specific projections of future percentage habitat loss (red), habitat gain (blue), and net habitat change (yellow) for *C. burnetii* under two climate change scenarios (SSP2-4.5 and SSP5-8.5), across five Global Circulation Models: ACCESS, IPSL, MIROC, MPI-ESM, and UKESM. Results are shown for the years 2050, 2070, and 2090.

**Fig. 5:**
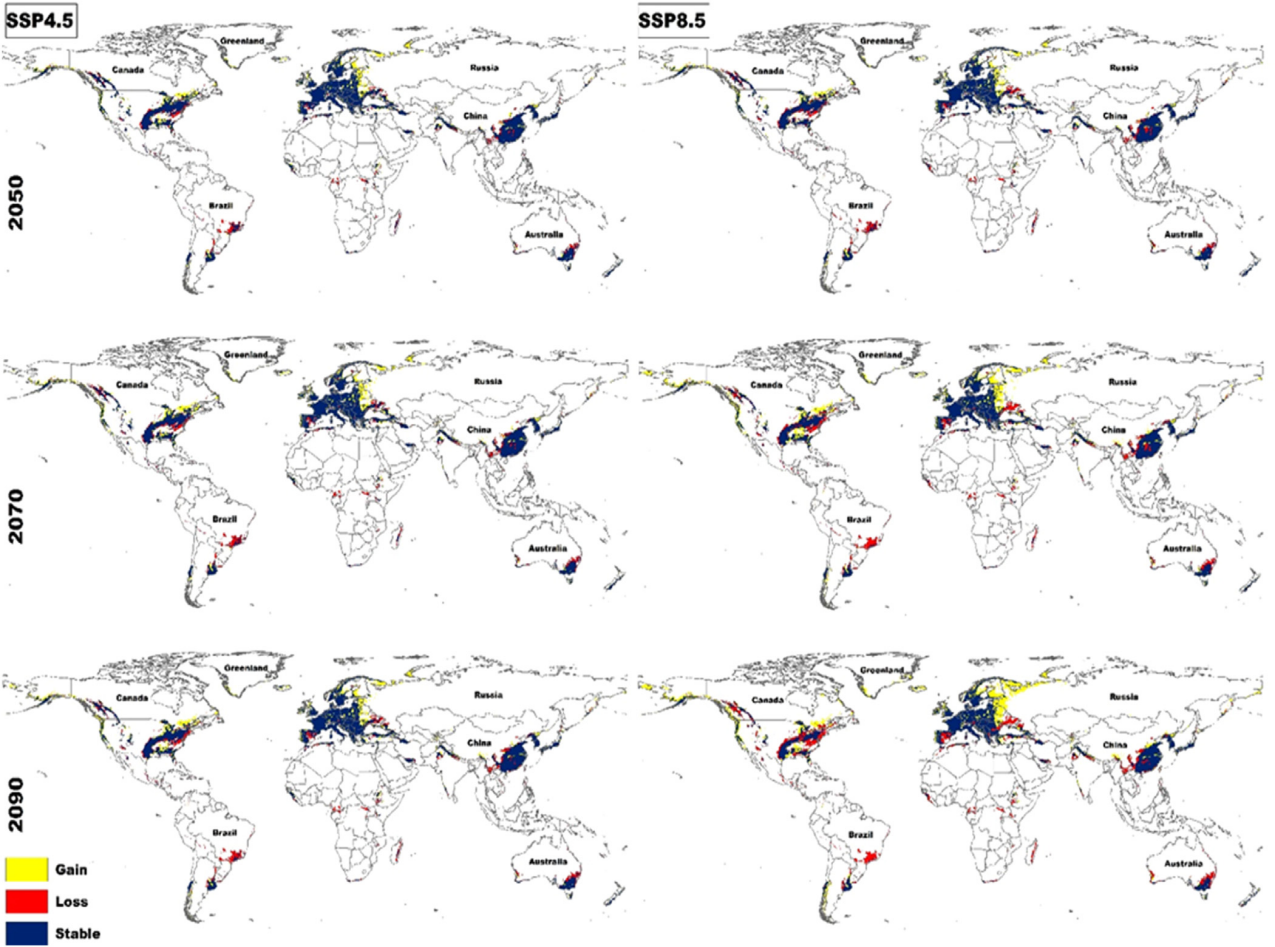
Projected changes in habitat suitability for *C. burnetii* under future climate change scenarios (SSP2-4.5 and SSP5-8.5) for the years 2050, 2070, and 2090. Areas of habitat gain are shown in yellow, habitat loss in red, and unchanged habitats in blue.

The results of our analysis indicate notable shifts in habitat suitability for *C. burnetii* under both moderate (SSP2–4.5) and severe (SSP5-8.5) climate scenarios across the future timeframes of 2050, 2070, and 2090. Generally, our models projected increased habitat suitability, particularly under the IPSL and ACCESS models, which showed the most substantial gains. In contrast, the UKESM and MPI-ESM models exhibited smaller increases in habitat suitability compared to the others (Fig. 4). These projections suggest that, over time, *C. burnetii* will likely spread to new areas, particularly under more extreme climate change conditions (SSP5-8.5 scenario). This northward expansion of habitat suitability into regions such as USA, Canada, and various parts of Europe, especially in the eastern region and Russia is probably driven by increases in temperature seasonality (bio4), which reflects more significant variability in temperature across seasons or rising precipitation in previously drier areas (bio14). Conversely, declining habitat suitability in regions like Australia, Brazil, Madagascar, and areas of central Africa could be linked to increasing aridification and reduced precipitation during the driest months, which limit the pathogen's ability to persist in these areas (Fig. 5).

By 2050, under the severe SSP5-8.5 scenario, habitat suitability is projected to increase by 24.97 % (range: 14.88–36.57 %) across the models, with a corresponding loss of 18.63 % (range: 15–20.8 %) of current suitable areas. By 2070, the gain in suitable habitats is expected to reach 34.7 % (range: 22–46.9 %), with a loss of 22.73 % (range: 21.4–25.1 %). By 2090, suitable areas are projected to increase by 44.56 % (range: 33–57.9 %), while 27.66 % (range: 22.4–31.7 %) of current habitats are expected to be lost (Fig. 5).By 2050, based on the moderate SSP2-4.5 scenario, habitat suitability is expected to increase by an average of 22.28 % (range: 16.63–31.65 %) across the models, with a projected loss of 15.70 % (range: 12.21–17.78 %) of current suitable areas. By 2070, the increase in suitable habitats is projected to reach 26.15 % (range: 17.90–35.90 %), accompanied by a loss of 17.42 % (range: 14.90–19.00 %). By 2090, suitable areas are expected to increase by 28.28 % (range: 18.29–37.28 %), while 19.40 % (range: 16.67–20.71 %) of current habitats are predicted to be lost.

## 4 Discussion

This study represents a significant advancement in understanding the potential impacts of climate change on *C. burnetii's* global distribution. For the first time, we provide a comprehensive, ensemble-based prediction of the pathogen's habitat suitability, encompassing multiple timeframes (2050, 2070, and 2090) and using a wide range of global circulation models (GCMs) under two distinct climate change scenarios. By leveraging an ensemble modelling approach that integrates multiple SDMs and GCMs, our findings offer excellent predictive reliability and comprehensive geographic insights into future habitat shifts.

The global distribution of *C. burnetii* encompasses North America (particularly the eastern USA), South America (e.g., Brazil), Europe, Africa (e.g., Guinea, Kenya, Ethiopia), Asia (e.g., China, Pakistan), and Australia. These distributions have been confirmed through various research studies, including in the United States [63], China [64], Pakistan [65], Italy [66], France [67], The Netherlands [68], and Algeria [69]. Additionally, [70] identified *C. burnetii* coinfections in the western hemisphere, particularly in the United States and Brazil, as well as in central Europe and Asia.

The SDMs results for *C. burnetii* demonstrated excellent predictive accuracy for all models. Our analysis showed that RF had the highest predictive performance, followed closely by the gradient boosting machine (GBM) and MaxEnt models. In contrast, the generalized linear model (GLM) had the lowest predictive performance [43], using only the MaxEnt model, reported strong results, although not as strong as those achieved by RF and GBM in our study. This comparison suggests that while MaxEnt is a reliable tool for SDMs, advanced ensemble models such as RF and GBM may provide superior performance in predicting the distribution of *C. burnetii*.

Our results revealed that temperature seasonality (bio4) and precipitation of the driest month (bio14) were the most important variables in predicting the distribution of *C. burnetii*. These findings are consistent with previous research, which also identified precipitation of the driest month (bio14) as a critical predictor of habitat suitability for *C. burnetii* [43,44]. In another study conducted in Greece, precipitation of the wettest quarter (bio16) and precipitation of the wettest month (bio3) were found to be the most significant contributors to the distribution model [71].

The significance of these variables may be explained by the fact that areas with low precipitation and high temperatures provide ideal conditions for the wind-borne dissemination of *C. burnetii*, which is a key transmission route for the pathogen [72]. The importance of wind in transmitting *C. burnetii* has been confirmed in previous studies [73,74]. Additionally, as an obligate intracellular pathogen, *C. burnetii* forms endospore-like structures, allowing it to survive for extended periods in hot and dry environments. This further enhances its persistence and spread in these conditions.

Future projections under the SSP2-4.5 and SSP5-8.5 climate change scenarios indicate significant shifts in the global habitat suitability for *C. burnetii*, emphasizing the profound impact of climate change on the environmental factors that influence the pathogen's survival and transmission dynamics. By 2090, our findings demonstrated a significant northward expansion of suitable habitats into northern Europe, Russia, and Canada, particularly under the severe SSP5-8.5 scenario. These regions, which are currently less impacted by *C. burnetii*, could become significant hotspots in the coming decades, underscoring the need for proactive public health measures to mitigate potential outbreaks.

Additionally, we observed increases in habitat suitability across Europe, China, and the United States. These regions, currently identified as high-risk zones for *C. burnetii*, are projected to remain stable and expand as climatic conditions become increasingly suitable for the pathogen's persistence. Notably, China has a reported seropositivity of 10 % [75], which could rise as habitat suitability expands. Similarly, Turkey, where human seroprevalence ranges 12.3–32 % [76,77], is expected to experience further habitat gains. Spain, with a seroprevalence of 15.3 % [78], is projected to face a decline in suitable habitats, whereas Denmark (with an 11 % seroprevalence) [79] is expected to maintain relatively stable habitat suitability. Therefore, preventive measures are needed for these regions.

In contrast, declines in habitat suitability are projected for regions such as South America, Australia, and parts of Africa. While these areas currently represent significant risk zones for *C. burnetii*, they may become less suitable due to increasing aridification and alterations in precipitation regimes. Considering the high current seroprevalence estimates in Australia and the lack of significant difference in infection rates between rural and metropolitan populations [80], as well as the presence of *C. burnetii* in wild rodents living near human dwellings in Brazil [81], these reductions in habitat suitability probably present a potential opportunity to reduce Q fever in these regions. Nevertheless, these projected reductions should be interpreted cautiously, as anthropogenic factors, including socio-economic conditions, human mobility, and global livestock trade, could still facilitate sporadic outbreaks, even in regions where habitat suitability is expected to decline.

The methods and findings presented in our study provide a valuable foundation for understanding how climate change may influence the distribution and dynamics of various diseases on a global scale. Species distribution modelling (SDM) approaches offer powerful tools for projecting the future suitability of pathogens under different climate scenarios. These models are not limited to zoonotic pathogens like *C. burnetii*; they can also be applied to pathogens affecting crops and humans, allowing for a comprehensive assessment of climate-driven risks. Despite their potential, studies examining the effects of climate change on infectious disease distributions remain limited, particularly at the global level [82,83]. By adopting a global approach, our study provides critical insights into these dynamics, helping identify large-scale patterns and emerging risk hotspots under future climate scenarios.

This study focused on climatic variables to predict habitat suitability. However, our models did not directly incorporate species interactions, human activities, and land-use changes. These factors can significantly influence habitat suitability. Moreover, although our analysis effectively identifies regions with suitable habitats for *C. burnetii*, it does not consider the complex disease transmission dynamics between animals and humans. Excluded variables such as livestock density, farming practices, and local health infrastructure may limit our predictions' precision and practical applicability. Incorporating these variables in future studies would likely enhance the accuracy and relevance of habitat suitability models for *C. burnetii*.

## 5 Conclusion

This study provides a comprehensive analysis of how *C. burnetii*'s habitat suitability may shift globally under future climate change scenarios. By employing an ensemble approach using SDMs and multiple GCMs, we were able to generate robust predictions of the pathogen's future distribution. Our hypothesis that climate change would drive a northward shift in *C. burnetii*'s habitat suitability while reducing suitability in more arid regions was confirmed. Specifically, projections under the SSP5-8.5 scenario revealed significant habitat expansion into northern regions such as Europe, Russia, and Canada, alongside a notable decline in suitability across parts of South America, Africa, and Australia.

These results emphasize the need for proactive public health strategies to manage emerging risks in newly suitable areas. While the decline in habitat suitability in regions such as South America and Australia may result in reduced Q fever incidence in these areas, these projections should be interpreted with caution. Anthropogenic factors, such as global livestock trade and human mobility, may still facilitate sporadic outbreaks even in regions with reduced habitat suitability.

Future studies should focus on incorporating additional factors such as land-use changes, livestock density, and human mobility patterns, which could further refine habitat suitability predictions and disease transmission dynamics. Furthermore, exploring potential mitigation strategies, such as vaccination campaigns in high-risk areas, and studying the socio-economic impacts of *C. burnetii's* habitat shifts, would provide valuable insights for public health planning.

## 6 Generative AI statement

The authors affirm that no generative AI was employed in generating the methods, codes, modelling results, figures, or tables presented in this work. Instead, AI tools (ChatGPT) and Grammarly were utilized solely to improve the text's readability, style, and accuracy in grammar, spelling, punctuation, and tone. Following the use of the tool, the authors thoroughly reviewed and edited the content as needed and take full responsibility for the content of the published article.

## Funding

This project was supported by an internal grant from Qatar University (CG24/25–383).

## CRediT authorship contribution statement

**Abdallah Falah Mohammad Aldwekat:** Writing – original draft, Data curation. **Niloufar Lorestani:** Writing – review & editing, Writing – original draft, Visualization, Validation, Supervision, Software, Methodology, Investigation, Formal analysis, Data curation, Conceptualization. **Farzin Shabani:** Writing – review & editing, Writing – original draft, Visualization, Validation, Supervision, Software, Resources, Project administration, Methodology, Investigation, Formal analysis, Data curation, Conceptualization.

## Declaration of competing interest

The authors declare that the research was conducted in the absence of any commercial or financial relationships that could be construed as a potential conflict of interest.

## Appendix Supplementary materials

Image, application 1
