# Supplementary material for: Impacts of climate change on the global spread and habitat suitability of Coxiella burnetii: Future projections and public health implications
Source: J Clim Chang Health. 2025 Apr 15;22:100442. doi: 10.1016/j.joclim.2025.100442 (PMC12851218; doi:10.1016/j.joclim.2025.100442)
Supplement: Supplementary file 1 [file mmc1.docx]

**Table S1. Detailed references of georeferenced maps of *Coxiella burnetii* prevalence across various countries.**

This table presents the georeferenced sources of *Coxiella burnetii* prevalence data collected from various countries and studies. The species presence points extracted from these maps were used in this research to analyze geographic distribution patterns.

| **Country** | **MAP/Reference Source** |
| --- | --- |
| **Australia** | <http://doi.org/10.1371/journal.pone.0233100> |
| **Australia** | <http://doi.org/10.1016/j.prevetmed.2021.105282> |
| **Belgium** | <http://doi.org/10.3389/fcimb.2020.625576> |
| **Brazil** | <http://doi.org/10.1016/j.bjid.2020.05.003> |
| **Brazil** | <https://doi.org/10.1093/trstmh/trab113> |
| **China** | <http://doi.org/10.1017/S0950268814002593> |
| **Denmark** | <https://doi.org/10.1186/s13028-014-0046-2> |
| **Greece** | <https://doi.org/10.3390/pathogens10030287> |
| **Guinea** | <https://doi.org/10.3390/microorganisms11061433> |
| **Hungary** | <http://doi.org/10.1186/1746-6148-10-107> |
| **Hungary** | <http://doi.org/10.1177/1040638714563566> |
| **Italy (central)** | <http://doi.org/10.1017/S0950268819002115> |
| **Italy (northwest )** | <http://doi.org/10.1016/j.prevetmed.2016.05.014> |
| **Kenya** | <http://doi.org/10.1155/2022/3741285> |
| **Netherlands** | <http://doi.org/10.1186/1746-6148-7-81> |
| **Spain (Northeastern)** | <http://doi.org/10.1017/S0950268812000271> |
| **Spain (Northeastern)** | <https://doi.org/10.1186/s13028-018-0429-x> |
| **Spain** | <https://doi.org/10.3390/ani14050749> |
| **Pakistan** | <https://doi.org/10.1016/j.actatropica.2016.07.017> |
| **Quebec** | <http://doi.org/10.1017/S0950268821001412> |
| **Réunion Island** | <http://doi.org/10.1371/journal.pntd.0003055> |
| **South Korea** | <https://doi.org/10.1016/j.prevetmed.2024.106157> |
| **Sweden** | <http://doi.org/10.1186/1751-0147-56-39> |
| **Switzerland** | <http://doi.org/10.1111/tbed.12362> |
| **Turkey** | <https://journals.lww.com/jvbd/fulltext/2017/54020/>  the_seroprevalence_of_coxiella_burnetii_in.6.aspx |
| **USA** | <http://doi.org/10.1186/1471-2180-14-41> |
| **Australia (website)** | Ponder W. F., Hallan A., Shea M. E., Clark S. A., Richards K., Klunzinger M. W., and Kessner V., 2023. Australian Freshwater Molluscs, Revision 2. |
